# Supplementary material for: Season of prescribed burns and management of an early successional species affect flower density and pollinator activity in a pine savanna ecosystem
Source: PeerJ. 2022 Nov 11;10:e14377. doi: 10.7717/peerj.14377 (PMC9661972; doi:10.7717/peerj.14377)
Supplement: Supplemental Information 1 [file peerj-10-14377-s001.docx]

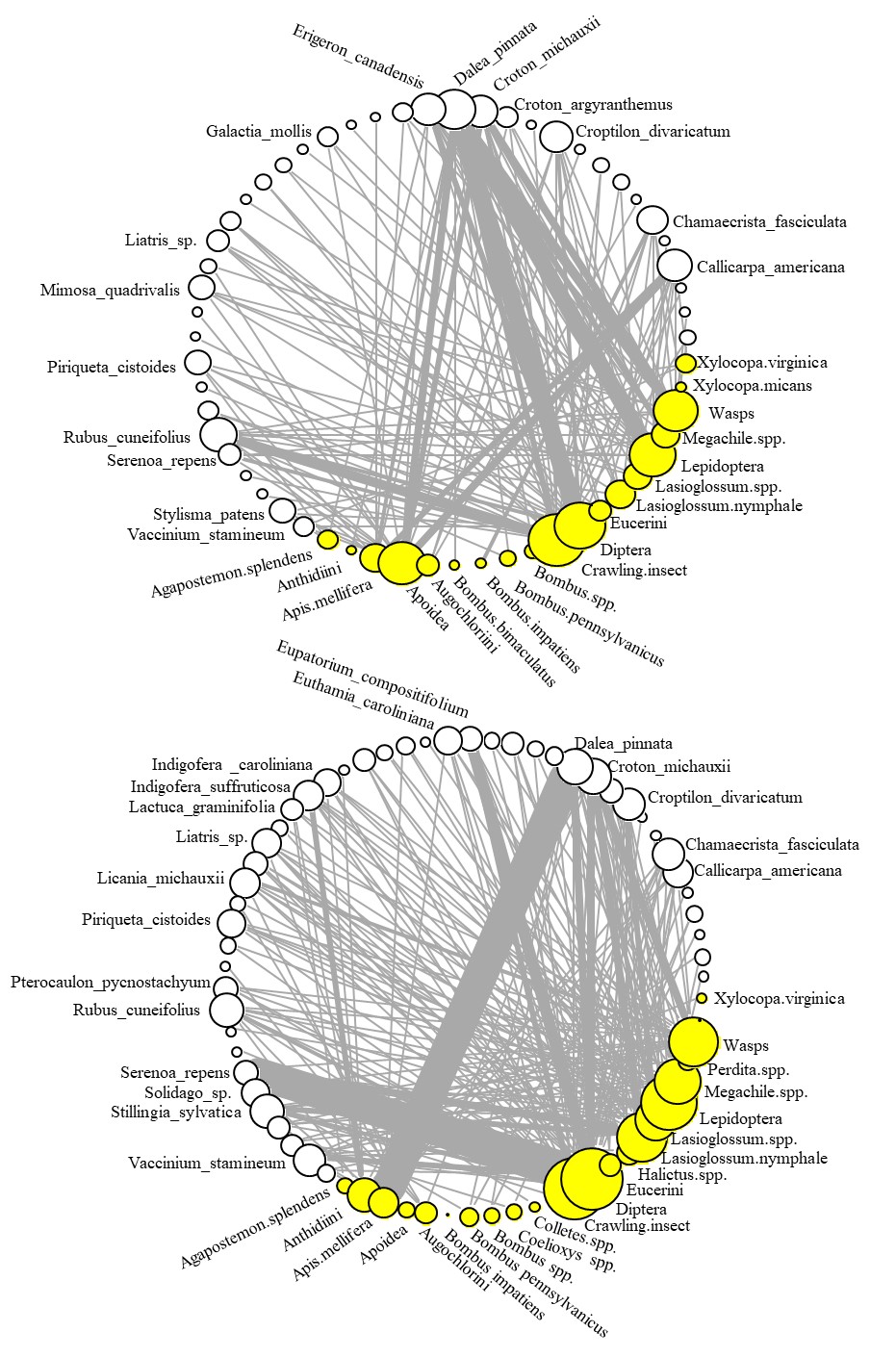


B

A

Appendix 1: Bipartite network plot showing plant-pollinator interactions in (A) 2019, the year between seasonal burn treatment and (B) 2020, the year when burn treatments were again applied
